# Inulin-Coated ZnO Nanoparticles: A Correlation between Preparation and Properties for Biostimulation Purposes

**DOI:** 10.3390/ijms25052703

**Published:** 2024-02-26

**Authors:** Lorenzo Gontrani, Elvira Maria Bauer, Lorenzo Casoli, Cosimo Ricci, Angelo Lembo, Domenica Tommasa Donia, Simone Quaranta, Marilena Carbone

**Affiliations:** 1 Department of Chemical Science and Technologies, University of Rome Tor Vergata, Via della Ricerca Scientifica 1, 00133 Rome, Italy; lorenzo.gontrani@uniroma2.it (L.G.); lorenzo.casoli@uniroma2.it (L.C.); cosimo.ricci@uniroma2.it (C.R.); angelo.lembo@uniroma2.it (A.L.); dodomemy@gmail.com (D.T.D.); 2 Institute of Structure of Matter-Italian National Research Council (ISM-CNR), Strada Provinciale 35d, n. 9, 00015 Monterotondo, Italy; elvira.bauer@ism.cnr.it; 3 Institute for the Study of Nanostructured Materials-Italian National Research Council (ISMN-CNR), Strada Provinciale 35 d, n. 9, 00010 Montelibretti, Italy; simone.quaranta@cnr.it

**Keywords:** ZnO nanoparticles, preparation methods, structural properties, electronic properties, biostimulation

## Abstract

Within the framework of plant biostimulation, a pivotal role is played by the achievement of low-cost, easily prepared nanoparticles for priming purposes. Therefore, in this report, two different synthetic strategies are described to engineer zinc oxide nanoparticles with an inulin coating. In both protocols, i.e., two-step and gel-like one-pot protocols, nanoparticles with a highly pure ZnO kernel are obtained when the reaction is carried out at T ≥ 40 °C, as ascertained by XRD and ATR/FTIR studies. However, a uniformly dispersed, highly homogeneous coating is achieved primarily when different temperatures, i.e., 60 °C and 40 °C, are employed in the two phases of the step-wise synthesis. In addition, a different binding mechanism, i.e., complexation, occurs in this case. When the gel-like process is employed, a high degree of coverage by the fructan is attained, leading to micrometric coated aggregates of nanometric particles, as revealed by SEM investigations. All NPs from the two-step synthesis feature electronic bandgaps in the 3.25–3.30 eV range in line with previous studies, whereas the extensive coating causes a remarkable 0.4 eV decrease in the bandgap. Overall, the global analysis of the investigations indicates that the samples synthesized at 60 °C and 40 °C are the best suited for biostimulation. Proof-of-principle assays upon *Vicia faba* seed priming with Zn5 and Zn5@inu indicated an effective growth stimulation of seedlings at doses of 100 mgKg^−1^, with concomitant Zn accumulation in the leaves.

## 1. Introduction

Nano-priming has a pivotal role in sustainable agriculture [1,2,3], since it is a pillar in ameliorating the indicators of plant well-being, improving their germination rate and seedling and plant growth [4], and alleviating abiotic climate and contamination as well as biotic stresses [5]. Besides the longtime recognized properties of nanomaterials in the field of sensoristics as carbon/quantum dots [6,7,8,9,10,11,12,13,14,15,16,17,18,19], as oxide or metal nanoparticles [20,21,22,23,24,25,26,27,28,29,30,31,32,33,34], in electronic devices [35,36,37,38,39,40,41,42,43,44,45,46,47,48,49], as well as in antimicrobic treatments [50,51,52,53,54,55,56,57,58,59,60,61], they are finding use, to an increasing extent, in agriculture [62,63,64,65,66,67]. Here, they may enhance productivity, involving gradual release and a proper pesticide dosage to aid pest control. Furthermore, they may be applied to seedlings or their parts, as well as young plants in active growth phases, allowing the amelioration/evaluation of defense responses in specific plant regions or organs, for instance, the root system [68,69]. In this regard, a large variety of nanoparticles were employed, aiming to improve the biometric parameters as well as micronutrient deficiencies [70,71,72,73,74,75]. Among the possible nanomaterials, ZnO nanoparticles (NPs) are widely used as nano-priming agents, since they can tolerate Zn soil deficiency, promote plant well-being, and can be produced at a low cost [76,77,78,79,80,81,82]. Additional improvements in plant growth and well-being can be achieved by employing suitably coated NPs, where the coating acts as a mimicking agent to facilitate entry via the roots and transport through the plant. This is the case, for instance, for Fe_3_O_4_ NPs coated with *Cassia occidentalis* L. flower extract [83], Au NPs whose absorption was implemented by galanga rhizome extract [84], or Ag coated with layers of alfalfa extract [85]. As for ZnO NPs for biostimulation purposes, currently explored coatings are cysteine [86], chitosan [87], or lignin [88].

**Figure 1:**
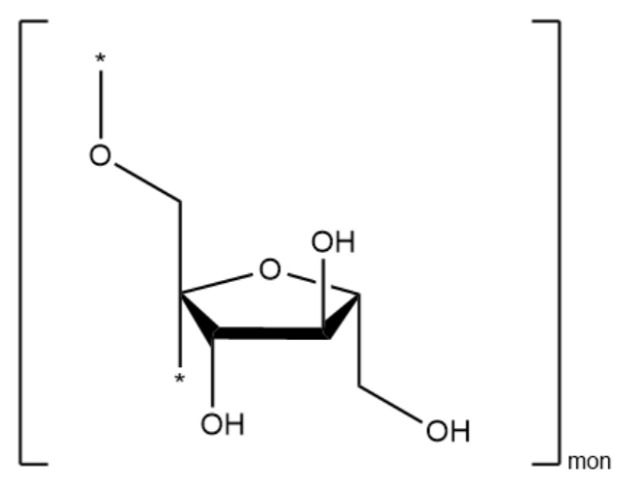
A sketch of the inulin monomer. Asterisks mark the connection points.

One of the latest biostimulants employed for *Vicia faba* L. seedling growth includes ZnO NPs coated with inulin [89], a fructan, as sketched in Figure 1. This can be extracted from several plants such as chicory roots (*Cichorium intybus* L.), Jerusalem artichoke tubers (*Helianthus tuberosus* L.), or novel sources like globe artichoke inflorescence (*Cynara cardunculus* L.) [90], as well as from agave (*Agave sisalana* P.) [91].

Size, morphology, the degree of coating, and the overall chemico-physical properties of the nanoparticles play a pivotal role in the efficacy of their action as biostimulants. Therefore, the synthetic strategy becomes crucial for the ensuing application. These combined properties, however, are often overlooked when dealing with plant administration, often relying on commercial nanoparticles that are not always fully characterized [75,92,93,94,95,96,97,98]. Engineered nanoparticles for biostimulation are usually lab prepared, although the implementation of the protocol is typically not discussed. Requirements of coated nanoparticles are uniformity in size and a sufficient degree of coating, as well as being low-cost and simple to prepare.

In the current paper, we focus on different synthetic pathways to achieve inulin-coated ZnO NPs. In particular, two different approaches were explored, i.e., two-step and gel-like processes. The resulting NPs were characterized by different techniques aimed at determining differences in the structural, morphological, and electronic properties, the latter being especially important because ZnO NPs act on the antioxidant potential in seedlings [99]. As for the two-step procedures, depending on the synthesis conditions Zn(OH)_2_ could be detected, whereas the temperature of the preparation phases could influence the size, morphology, and agglomeration of the particles, though not the bandgap. This is similar for all coated samples and in line with their uncoated counterparts. The gel-like process yields larger agglomerates (on average 300 nm) of smaller nanoparticles (on average 30 nm), characterized by a significant decrease in the bandgap. Overall considerations of the uniformity of the particle sizes, the degree and uniformity of the coating, and a not unusual bandgap point identified sample Zn5@inu as the best suited for further biostimulation application. Proof-of-principle assays upon *Vicia faba* seed, priming with Zn5 and Zn5@inu, indicated an effective growth stimulation of seedlings at doses of 100 mgKg^−1^ [89], with concomitant Zn accumulation in the leaves. In particular, verification in relation to whether the selected coated and non-coated samples had geno- or cytotoxic effects on *Vicia faba* seedlings was undertaken. Furthermore, preliminary experiments on single-step synthesized samples did not yield equally promising results in terms of biostimulation, pointing to the double-step synthesis as the best strategy for the effective sampling of the nanoparticles. Since the major differences between one- and two-step processes are related to the morphology and electronic properties of the samples, their influence on the biostimulation process has also been considered.

## 2. Results and Discussion

The synthesized samples were subjected to multiple characterization techniques to determine the phase, morphology, and degree of coverage.

### 2.1. XRD

**Figure 2:**
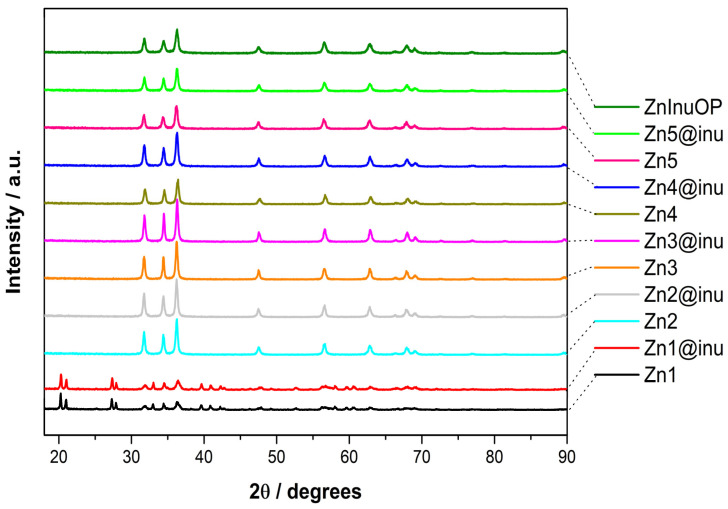
XRD patterns of the prepared samples, with and without coating. Non-coated: Black = Zn1, cyan = Zn2, orange = Zn3, brown = Zn4, pink = Zn5; coated: red = Zn1@inu, gray = Zn2@inu, magenta = Zn3@inu, blue = Zn4@inu, light green = Zn5@inu, dark green = ZnOInuOP.

The XRD patterns of the samples achieved with both procedures are reported in Figure 2. As for the two-step procedure, the XRD patterns achieved with and without an inulin coating are presented. The overall comparison of the spectra points at four main features: (i) ZnO is the main product, (ii) additional Zn(OH)_2_ may be present depending on the preparation conditions, (iii) inulin coating, as commonly happens in organic phases, is not singled out by XRD [100] due to the limited scattering power of the thin layer composed of light atoms, and iv) capping the NPs with inulin does not have an evident effect on the composition of the core, i.e., Zn(OH)_2_ is not removed by the inulin coverage.

In more detail, the curves shown in Figure 2 can be grouped into two clusters, whose components share the same global common features. In the top array of XRD patterns, the presence of a ZnO hexagonal phase (P6_3_mc space group, found naturally in the mineral zincite [101]) appears evident in every curve of the group from the very characteristic feature of that crystal lattice, i.e., the peak triad at 32, 34.5, and 36 degrees, corresponding to the reflections from 100, 102, and 101 crystallographic planes of the crystal system. Regarding the intensity of the triad peaks, the latter appears more intense in the three coated systems, Zn3@inu, Zn2@inu, and Zn4@inu (in decreasing intensity order), possibly signaling the existence of slightly larger crystal domains in the system. In the lower group of curves containing Zn1/Zn1@inu patterns (two elements), several new peaks can be pinpointed. Notably, two doublet peaks at 20–21 and 27–28 degrees appear, as well as a peak at 33 and low-intensity triplet peaks at 39, 41, and 42.5 degrees. These peaks can be attributed to the reflections of a zinc hydroxide orthorombic (ε) crystal phase naturally found in the mineral Wulfingite [102,103]. The two doublets, which have a stronger intensity than the other peaks of the pattern, are originated from the reflections of 011, 002, 111, and 102 crystal planes of the orhorombic lattice.

### 2.2. IR

**Figure 3:**
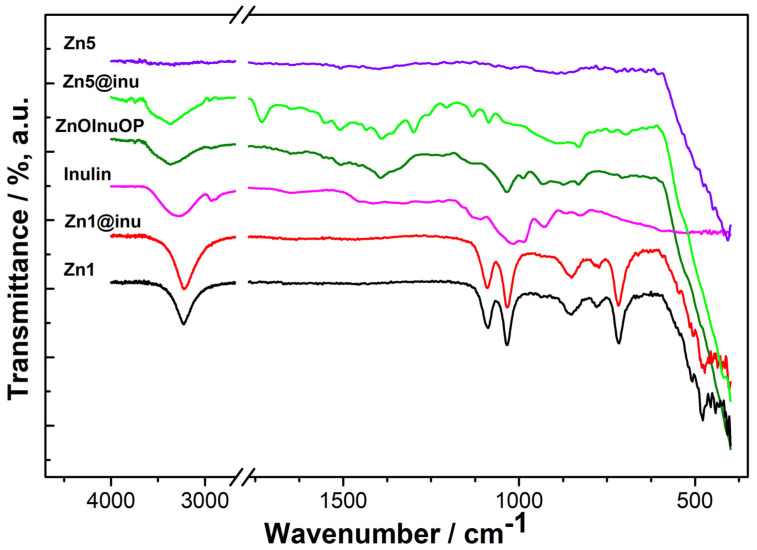
IR spectra of selected samples: violet solid line = Zn5, light-green solid line = Zn5@inu, dark-green solid line = ZnOInuOP, pink solid line = inulin, red solid line = Zn1@inu, black solid = line Zn1.

IR spectra may provide complementary data with respect to XRD patterns, i.e., information on the coating of the synthesized samples. Figure 3 displays spectra of selected samples, i.e., samples Zn1 and Zn1@inu containing Zn(OH)_2_, the one-pot sample ZnOInuOP and inulin-coated sample Zn5@inu showing inulin-related features, and the corresponding uncoated sample Zn5.

The Zn1 sample displays typical features of Zn(OH)_2,_ i.e., sharp and intense vibrational peaks around 3280 and 716 cm^−1^ associated with O−H stretching and bending modes. The presence of zinc hydroxide is further confirmed by the characteristic absorption for Zn−O−Zn asymmetric stretchings located at 1085 and 1030 cm^−1^ and Zn−O lattice vibrations at around 485 cm^−1^ [104]. Zn1@inu displays similar features to Zn1, thus indicating a coating of the particles below the detection limit. At variance with this, both coated samples Zn5@inu (two-step procedure) and ZnOInuOP (one-pot procedure) present features ascribable to inulin layers covering the nanoparticles. For comparison purposes, the spectrum of the inulin used for the preparation is also reported in Figure 3. The spectral features of inulin may be divided into three regions. The first one, at low wavenumbers in the 600–800 cm^−1^ range, is assigned to -CH_2_ rocking vibrations. The region between 800 cm^−1^ and 1400 cm^−1^ carries the inulin fingerprints. In particular, peaks at 827 cm^−1^ and 866 cm^−1^ are representative of the anomeric bendings δ(C1–H) and ring vibration of 2-ketofuranose, respectively, and are indicative of the β-(2→1) glycosidic bonds. In contrast, peaks at 1016 cm^−1^, 1109 cm^−1^, and 1219 cm^−1^ are assigned to the stretching bands of the fructose–furan ring [105]. A weak band at 1653 cm^−1^ is attributed to -OH bending of residual water. The broad band at 3304 cm^−1^ is due to the –OH stretching vibrations of inulin and residual water, and it is broader and shifted as compared to Zn1 due to the additional contributions.

The IR spectrum of ZnOInuOP is similar to that of inulin with some additional features, most notably the steep increase at 400 cm^−1^ due to the Zn-O asymmetric stretching. A larger amount of bands are observed in the 600–800 cm^−1^ range due to the interaction with ZnO, which causes additional -CH_2_ rocking vibrations. Furthermore, a small shift in the fructose–furan ring stretching to 1034 cm^−1^ and 1132 cm^−1^ is observed, which is also ascribable to the interaction with the ZnO nanoparticles. Additional bands are present at 1649 cm^−1^, 1556 cm^−1^, and 1508 cm^−1^, compatible with the chelation of Zn by two carbonyl groups (e.g., β-diketones), such as in Zn acetylacetonate [106]. These are an indication of the interaction between ZnO and inulin. The bending mode of residual water may contribute to the band at 1649 cm^−1^. Finally, the broad band centered at 3365 cm^−1^ is a convolution of several contributions to the asymmetric stretching from -OH groups of inulin, as well as residual water. A shoulder at 2932 cm^−1^ is assigned to C–H asymmetric stretching.

Since the synthesis of Zn5@inu is carried out in two steps, its IR features must be offset against the bands of the naked nanoparticles, i.e., Zn5, that are typical of pure ZnO, with a steep absorption at 400 cm^−1^ and a rather flat spectrum in the whole remaining absorption range.

The lack of evident ν_O–H_ vibrations in the region around 3300 cm^−1^ rules out the presence of residual water traces. Regarding Zn5@inu, a larger number of peaks is observed as compared to both inulin and ZnOInuOP in the 600–800 cm^−1^ range, thus indicating a more pronounced or a better-detected interaction with ZnO. Shifts are observed in the bands at 889 cm^−1^ and 829 cm^−1^, and two additional peaks are present at 1086 cm^−1^ and at 1205 cm^−1^. Furthermore, the relative intensity of the peaks in the 1000–1300 cm^−1^ region is opposite with respect to ZnOInuOP. In particular, Zn5@inu has stronger asymmetric C–C and C–O stretchings, whereas ZnOInuOP diplays a more intense C–O–C asymmetric stretching, possibly indicating a larger ring deformation in the latter case, due to a weaker coordination to ZnO. The bands at 1508 cm^−1^ and 1551 cm^−1^, assigned to the chelation of Zn by two carbonyl groups, are stronger than of ZnOInuOP. Furthermore, a strong band appears at 1730 cm^−1^, assigned to the stretching vibration of a carbonyl group that can be associated to an aliphatic ketone, an aldehyde, or an alfa-beta unsaturated ester. The combination of these observation points at a more pronounced chelation effect as compared to ZnOInuOP. A broad band at 3365 cm^−1^ is due to the contributions of asymmetric stretching from the -OH groups of inulin, as well as residual water. Finally, samples Zn2, Zn3, and Zn4 indicate the presence of ZnO, whereas Zn2@inu, Zn3@inu, and Zn4@inu all have similar spectra to Zn5@inu, though all features are less intense, hinting at a lower coverage by inulin.

The IR bands of the samples along with their assignment are reported in Table 1.

### 2.3. SEM

**Figure 4:**
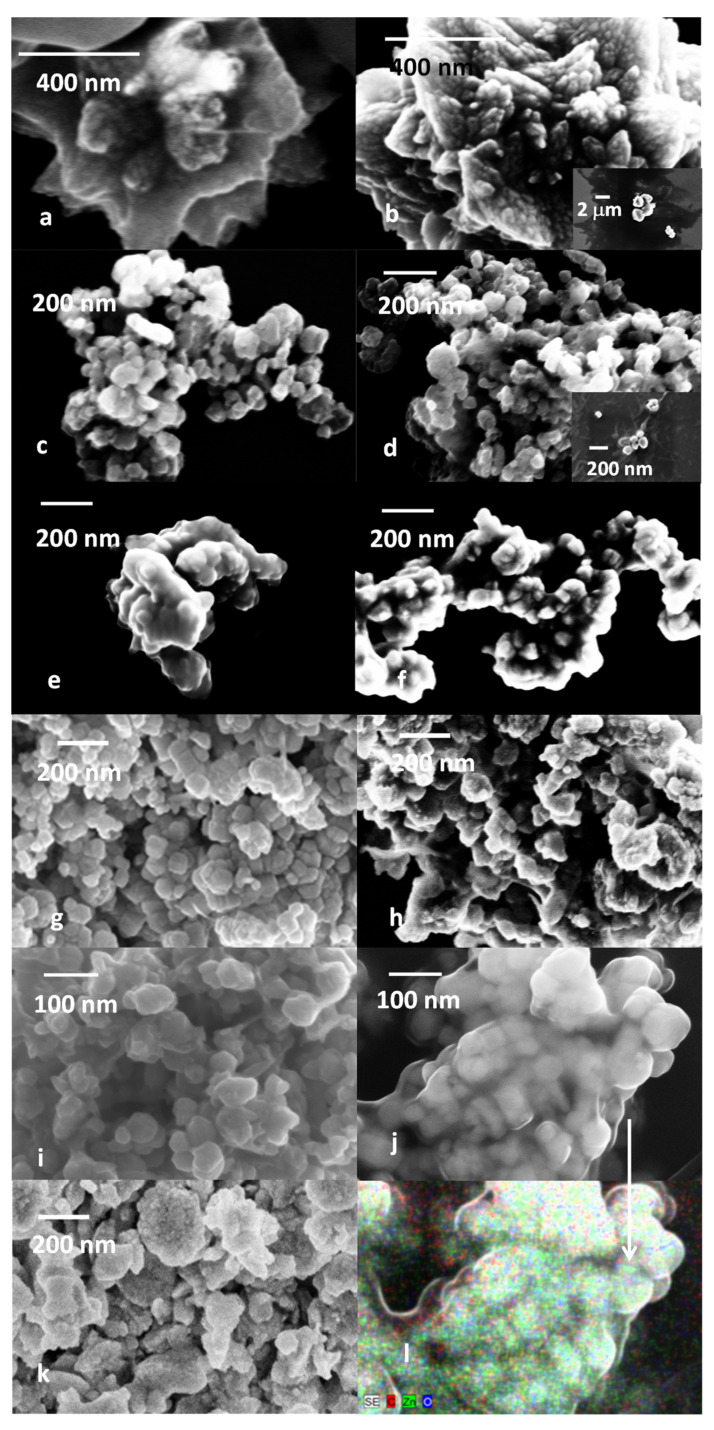
SEM images of the synthesized samples: (**a**) = Zn1, (**b**) = Zn1@inu, (**c**) = Zn2, (**d**) = Zn2@inu, (**e**) = Zn3, (**f**) = Zn3@inu, (**g**) = Zn4, (**h**) = Zn4@inu, (**i**) = Zn5, (**j**) = Zn5@inu, (**k**) = ZnOInuOP, (**l**) = EDS overlapped maps of Zn (green), O (blue), and C (red) of Zn5@inu.

The SEM images were analyzed in terms of the shape of the particles, average size, and size variation upon coating with inulin (for samples prepared according to the two-step procedure). In addition, EDX analysis was carried out to determine whether the coating procedure was successful. The SEM images are reported in Figure 4a–k, whereas Figure 4l is an EDS map of sample Zn5@inu.

In more detail, the sample Zn1 (Figure 4a) shows the characteristic flower-like shape of Zn(OH)_2_ [24,107] with an average size in the micrometer range, that is retained upon inulin coating (Figure 4b—Zn1@inu). Furthermore, a layer of coating can be observed in the inset at low magnification, whose analysis reveals the sole presence of C and O; hence, it was attributed to an excess of inulin. Samples Zn2, Zn3, Zn4, and Zn5 are all characterized by the presence of nanoparticles of a hexagonal to round shape. The nanoparticles of Zn2 have an average diameter of 62 ± 4 nm. The subsequent coating does not affect the nanoparticles’ average diameter that remains at 63 ± 4 nm. The composition of Zn2@inu is rather poor on carbon, as compared to the other coated samples. However, extensive layers of carbonaceous material are observed all around the agglomerates, all in all, pointing at a partial degradation of the inulin. Samples Zn3 and Zn3@inu both have nanoparticles with a 60 ± 10 nm average diameter, inglobated in larger structures to form agglomerates. Sample Zn4 has nanoparticles with a hexagonal shape with an average size of 52 ± 5 nm. The overall appearance becomes more aggregated and the average size of the nanoparticles is 54 ± 5 nm. Zn5 nanoparticles are roundish and with average size of 65 ± 4 nm, that decreases to 58 ± 1 nm upon inulin coating. Finally, ZnInuOP, the one-pot-synthesis sample, is characterized by the formation of nanoparticles that aggregate in irregular shapes, with sizes in the range of 100–300 nm. The size of the nanoparticles is estimated to be 30 ± 10 nm.

**Figure 5:**
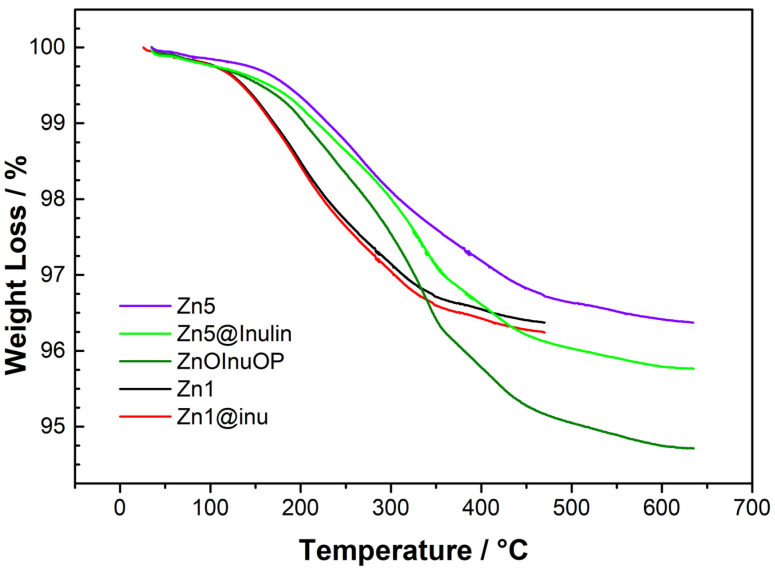
Weight losses of selected samples: violet solid line = Zn5, light-green solid line = Zn5@inu, dark-green solid line = ZnOInuOP, pink solid line = inulin, red solid line = Zn1@inu, black solid = line Zn1.

The shape, average size, and composition of the samples are reported in Table 2. Provided that the final goal of the preparation is the achievement of well-dispersed, well-coated nanoparticles, based on the analysis of SEM images, the most-suited preparation appears to be Zn5@inu; therefore, EDS mapping was carried out on this sample (Figure 5). The overlapped mapping of Zn, O, and C indicates a homogenous distribution of Zn, O, and C, thus indicating a uniform coating of inulin. A few considerations are in order in terms of the preparation method, based on the comparison of the images of all preparations. Although small, well-dispersed NPs are achievable at 40 °C, the coating at the same temperature does not yield a homogeneous layer, and rather, induces agglomeration. On the other hand, a full preparation at 60 °C yields a sample with a rather low carbon content, probably due to a mild denaturation. The best combination of temperature parameters of the first and second preparation stages, appears to be T_P_ = 60 °C, T_D_ = 40 °C. It must be noted that the sole synthesis of ZnO may provide small, regularly shaped nanoparticles [24]. However, the combination with the coating procedure requires higher starting temperatures. Finally, the one-pot preparation does yield samples that are well covered with inulin, but it is likely that a multi-layer is contributing to a large degree of agglomeration.

### 2.4. Thermogravimetric Analysis

The degree of coverage of the samples can be estimated through thermogravimetric analysis. The weight losses of selected samples are reported in Figure 5, whereas the weight loss differences between coated and uncoated pairs of samples are reported in Table 3. For sample ZnOInuOP, the difference was calculated with respect to Zn5.

As far as uncoated samples are concerned, they all present major losses of the order of 3.5% weight due to water absorption. A distinction must be made, however, between Zn1, i.e., the hydroxide and the ZnO samples, since the former loses weight in a narrower temperature range. Additional weight losses related to the inulin coatings range between 0.15% (Zn1@inu) and 1.67% (ZnOInuOP). These values have been used to estimate the average number of inulin monomeric units per particle, taking into account the average diameter of particles of each sample, as determined by SEM imaging. The estimate is accurate only for fairly separated nanoparticles, i.e., Zn5@inu, whereas it is more of an indication for agglomerates. In the calculation, the following formula was applied:

$${\mathrm{mass}}_{\mathrm{inulin}}=\frac{{\mathrm{mass}}_{\mathrm{uncoated}-\mathrm{NPs}}\;\cdot \;{\%\mathrm{WL}}_{\mathrm{inulin}}}{{\%\mathrm{WL}}_{\mathrm{uncoated}-\mathrm{NPs}}}\;$$

where mass_inulin_ = mass of inulin per particle; mass_uncoated-NPs_= mass of a ZnO nanoparticle or mass of a Zn(OH)_2_ particle, depending on the reference sample. Furthermore, it varies with the average diameter of the particle and it is for instance, 6.2 × 10^−16^ g, for the hexagonal closely packed wurtzite structure of ZnO with a 58 nm diameter. %WL_inulin_ = percentage weight loss due to inulin; %WL_uncoated-NPs_ = percentage weight due to residual particles. This leads to an average estimation of 4.6 × 10^−18^ g inulin per ZnO nanoparticle for the Zn5@inu sample that corresponds to 17,700 inulin monomeric units, or 590 polymeric units of 30 monomers each. Overall, the degrees of coverage are in line with the EDX measurements. The weight loss difference between Zn1@inu and Zn1 is rather small, as could be expected upon EDX analysis, indicating comparatively low C and O contents and corresponding to a coverage of 53,867 30-mers. Interestingly, the ratio between the coverage of the largest nanoparticle (Zn1@inu, d = 1200 nm) and the mean coverage of the two-step procedure samples (Zn5@inu, average d = 58 nm) is remarkably smaller than that expected by the surface increase, hypothesizing a one-layer coating or less. This feature hints at the establishment of a multi-layered coating in the smaller fragments.

### 2.5. Reflectance Spectroscopy

**Figure 6:**
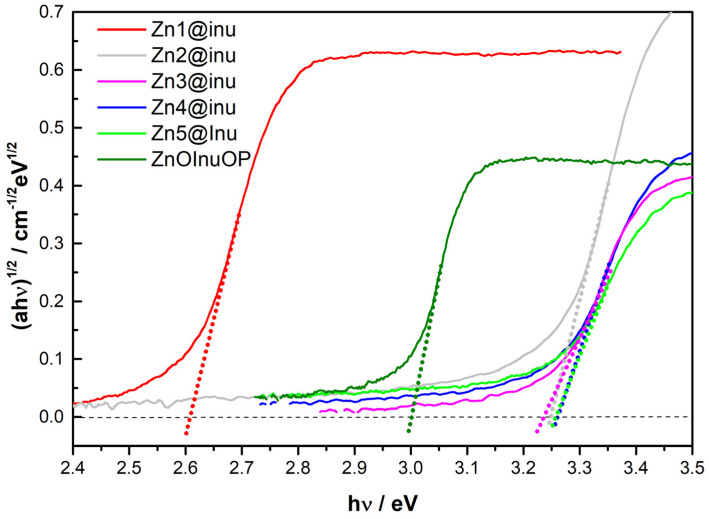
Tauc plots for the determination of optical bandgap E_g_ and associated extrapolations of the linear regions (dotted lines). Red = Zn1@inu, gray = Zn2@inu, magenta = Zn3@inu, blue = Zn4@inu, light green = Zn5@inu, dark green = ZnOInuOP.

Nanoparticles typically influence the redox properties of plants upon adsorption; therefore, an important issue is the determination of their electronic properties [108,109]. This was carried out using Diffuse Reflectance Spectroscopy (DRS), in particular, taking into account possible variations, correlated to the coating. In general, this technique is largely employed to estimate the electronic bandgap of solid materials, especially in the field of semiconductors, like the ZnO and Zn(OH)_2_ considered in this article. The DRS spectra of the synthesized samples are reported in Figure 6 (Tauc plots).

In particular, the reflectance spectra are associated with the absorption patterns by the Kubelka–Munk function [110,111], which states that the reflectance of an infinitely thick layer of sample ($R_{\infty })$ obeys the following formula:

$$\frac{K}{S}\equiv \frac{{\left(1-R_{\infty }\right)}^{2}}{2R_{\infty }}$$

in which, K and S are K-M absorption and scattering coefficients, respectively; the former parameter is related to the intrinsic absorption coefficient α by the equation α = *K*/2 [112], ultimately leading to *K/S* ≈ *α* whenever S can be considered constant across the various samples. Starting from the Kubelka–Munk absorption plot, the bandgap could be estimated by applying the Tauc relation [113]:

$$\alpha hv=C1{\left(hv-E_{g}\right)}^{n}$$

In this relation, the exponent values *n* = 1/2 and *n* = 2 apply to direct gaps (such as those of ZnO and Zn(OH)_2_ herein discussed) and indirect ones, respectively, with C1 being a constant. From the extrapolation of the linear part of the curve at the lowest energy side, corresponding to the energy value where α is zero (see Figure 6), the estimated bandgap values, which are reported in Table 4, could be obtained for the six inulin-coated samples. The reference values for bulk ZnO [114] and Zn(OH)_2_ [115] bandgaps are 3.37 and 2.88 eV, respectively.

As is clear from inspection of Table 4, the four samples obtained with the two-step protocol, which the IR and XRD studies demonstrated to be composed of (coated) ZnO, share approximately the same bandgap value, within the experimental and fitting errors. Such similarity complies with the analogous XRD patterns observed for Zn2@inu, Zn3@inu, and Zn4@inu. In contrast, the one-pot sample ZnOInuOP, still containing only ZnO and the coating (no hydroxide detected), shows a significantly smaller bandgap. In both cases, the values are smaller than bulk value (3.37 eV) reported in the literature. This is contrary to the generally expected bandgap opening effect upon size shrinking owing to the quantum confinement phenomena [116,117]. The sizes of uncoated ZnO nanoparticles were assessed with the same experimental procedure, and similar bandgap values (3.25–3.28 eV, i.e., smaller than bulk value) [24] were found. These correlated to the nanoparticle diameters observed in an SEM study (around 15 nm) with a modified version of the Brus formula [112]. Further and even more relevant effects can come into play when the coating is applied to the nanoparticles’ surface. For instance, it was found that the amount of coating can remarkably reduce the bandgap (up to 0.5 eV) of graphene-oxide-coated Mn_3_O_4_ nanoparticles [118]. However, recently, Guan et al. [119] showed that treatment of titania with carbon powder led to the formation of fiber-like structures on the TiO_2_ surface, with a sizable bandgap narrowing due to the formation or modulation of defects like oxygen vacancies (Ovs), onto the titania surface. Since similar mechanisms are thought to be crucial in modulating ZnO optoelectronic properties [120], it is not at all surprising that coatings, like those applied in the present study, may have a strong effect and reduce the bandgap by almost 0.4 eV in the one-pot coated sample. Therefore, the notable bandgap reduction in ZnInuOP may be due to a combination of effects, namely the coating effect just described and the aggregation of the (uncoated) nanoparticles, which have small dimensions individually (d ≈ 30 nm), into larger aggregates (hundreds of nm). Ultimately, this reduces the quantum confinement effect that the latter would bring about. Regarding Zn1@inulin, the only coated hydroxide considered in the analysis, it was found to have a 2.61 eV bandgap, thus showing a percentage reduction from the bulk value in line with that experienced by ZnInuOP, the other sample possessing extensive inulin coating.

### 2.6. Plant Growth Assays—Proof of Principle

**Figure 7:**
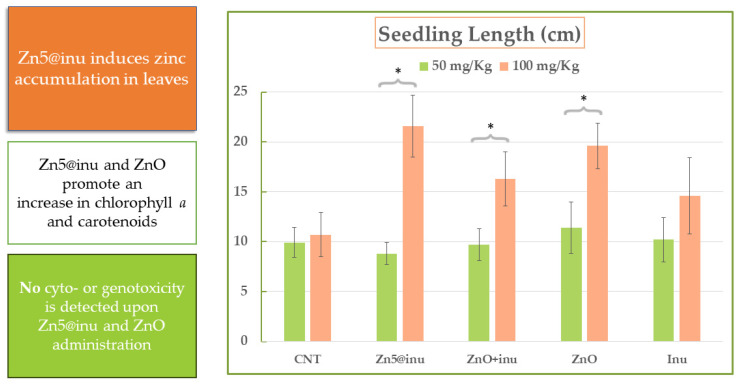
Summary of the biostimulation effects of Zn5@inu samples, from Ref. [89], with modifications. Asterisks identify tests with *p* < 0.05.

Plant growth tests were performed on the selected samples, i.e., Zn5@inu and its uncoated counterpart, in order to appraise the biostimulating effect as well as a possible adverse (cytotoxicity) response to the materials and eventually correlate the observed data with structural and optical response properties. The plants employed in the tests were *Vicia faba* L (faba bean) seedlings, and the quantity of ZnO absorbed by the different plant tissues was assayed through ED-XRF analyses [89], focusing on the fluorescence signal coming from the zinc metal atoms that was proportional to ZnO concentration. As proof of principle, two dosing levels were considered, namely low and high dose, i.e., 50 mg kg^−1^ and 100 mg kg^−1^, respectively. In detail, the measurements highlighted that the nanomaterial was thoroughly absorbed by the plant tissues. The increase in Zn level was actually significant in any case, with a larger relative accumulation in the leaves at the high dose and in the roots at the low dose. This corresponded to the increment ranges +289% to +9001% in the former and to +512% to +3704% in the latter case, for the two extreme cases, i.e., plantlets cultivated with ZnO + inu 50 mg kg^−1^ and with Zn5@inu 100 mg kg^−1^. Interestingly, the administration of inulin only (both at 0.3% and 0.6%) caused significant changes in the Zn content in the leaves but not in the roots with respect to the control seedling. The full table of ED-XRF results is reported in [89]. The germination tests carried out showed that no treatment induced a sizeable change in the seed germination percentage, except for the treatment with uncoated ZnO at the maximal dose; under these conditions, an increase in the germination rate was observed, suggesting a possible role in promoting sprout development of this compound. On the contrary, in the post-germination phase, the high accumulation of zinc, especially in plant leaves, seems to result in a marked effect on plant growth; indeed, the seedling dimensions were found to increase by an impressive +103% when Zn5@inu was administered, followed by a noteworthy +84% when the mixture ZnO + inu was supplied, and by a still noticeable  +53% when uncoated ZnO was given to the plant. Parallel to the high stimulating power, the zinc oxide/inulin composite shows an absence of toxicity/genotoxicity (trypan blue staining test [89]), even at the highest dose administered. In particular, for the former assessment (toxicity), very negligible differences were evidenced between each sample (coated and uncoated) and the control sample, independent of the amount supplied. A maximum cytotoxicity value of approximately 17% of the total area was reached, which was regarded as physiological, considering that 11% of the stained tissue was assessed in the CNT. Regarding the latter (genotoxicity), the frequency of micronuclei (MN) was evaluated, and comparable values between the negative control (CNT) and all the various Zn@inu-treated samples were found both after 24 h and 72 h of treatment, thus suggesting the absence of genotoxicity. This is at variance with a parallel experiment where the mutagenic herbicide maleic hydrazide, considered as the positive control, was supplied to the same plant specimens. The latter appraisal showed a marked MN frequency increase and confirmed *V. faba* sensitivity to mutagenic compounds. A further confirmation came from the absence of hydrogen peroxide, signaling that oxidative stress in the plant is very low. Finally, the NP treatment with Zn5@inu at a high dose led to a remarkable increase in chlorophyll a and b contents (+19% and +30%, respectively) in the seedlings, accompanied by an increase in carotenoid content (+22%), compared to the control sample. Treatment with ZnO led to slightly lower figures (+16% and +27%), whereas the opposite behavior was observed for the ZnO + inulin combination (no coating), scoring a decrease (−15%) in Chl b content. Overall, our findings reveal that the coated version of the prepared ZnO nanoparticles (maximally Zn5@Inu) can give a very large boost to plant growth in the post-germinative phase. The most plausible reason for this issue resides in the sugar coating, which renders the NPs’ internalization and transport across the plant tissues more efficient, thus promoting a massive metal migration towards the leaves once absorbed by the roots, while protecting against their degradation, and ultimately increasing their bioavailability in the periphery. The accumulation of ZnO NPs in the plant leaves can counteract the oxidative stress by acting as Reactive Oxygen Species (ROS) scavengers. Moreover, owing to the optoelectronic properties, which are almost fully conserved upon inulin coating, as evidenced by the study reported in Paragraph 2.5, the material may act as a spectrally selective system that optimizes the solar energy harvesting by the underlying leaf green pigments. This is carried out by filtering out the unusable portion of the solar spectrum and minimizing unwanted system overheating [121,122], with the inulin coating playing the role of the preferential carrier of the ZnO NPs through the apoplastic and symplastic pathways [123]. A summary of the biostimulation effects of Zn5@inu is reported in Figure 7 [89].

Biostimulation with ZnOInuOP at both doses was not very effective, since germination and seedling growth were similar to the control, within the error margin. This opens up a complete scenario on possible explanations. The major differences in ZnOInuOP with respect to Zn5@inu are the larger NP aggregation and the lower bandgap, which may interplay to yield a low level of biostimulation. On one hand, the large, coated aggregates might encounter difficulties entering the root sheaths, or being metabolized and transported to the leaves. On the other hand, the small bandgap might play a negative role in plant growth, in so far as the particles are absorbed and remain as a whole.

## 3. Materials and Methods

### 3.1. Chemicals and Equipment

Zinc nitrate hexahydrate (Zn(NO_3_)_2_▪6 H_2_O) and sodium hydroxide (NaOH) were purchased from Sigma-Aldrich (St. Louis, MI, USA), while the inulin was provided by ThermoFisher (Waltham, MA, USA).

XRD patterns were collected using an X’pert pro X-ray diffractometer by Philips, operated with CuK-alpha radiation. The scans were carried out with a 2θ step size of 0.02° and a 2 s/step counting time. FTIR spectra were obtained with a Shimadzu Prestige-21 instrument equipped with an attenuated total reflectance (ATR) diamond crystal (Shimadzu Ltd., Kyoto, Japan) in the range of 400–4000 cm^−1^, with a resolution of 4 cm^−1^.

SEM images were taken with a Zeiss Auriga Field-Emission Scanning Electron Microscope instrument SUPRA™ 35 (Carl Zeiss SMT, Oberkochen, Germany) operating at 6–8 kV. The EDX analysis was carried out, operating the SEM at 20 kV and coupling it with the EDS/EDX, using INCAx-sight, Model 7426 (Oxford Instruments, Abingdon, Oxfordshire, UK). The samples for SEM imaging were prepared either by a direct deposition on carbon tape or by drop-casting deposition of a powder dispersion in ethanol onto a silicon sample holder. Termogravimetric analysis (TGA) was also performed using a Q600 thermobalance instrument, in the presence of a dry N_2_ flow (100 mL min^−1^), with a ramp of 10 K min^−1^ from 300 to 1073 K. Ultraviolet–visible (UV–Vis) and near infrared (NIR) studies were performed using diffuse reflectance instrumentation by Avantes. The apparatus is composed of an integrating sphere (diameter 30 mm) coated with a highly reflective material (Spectralon^®^) that is illuminated by the radiation emitted by a halogen/deuterium source (AvaLight-DH-S-BAL-Labsphere, Lafayette, CO, USA), collimated by UV–Vis lenses, and introduced into the sphere by optical fiber wires. The sphere acts as a light collector, and is then used as a diffuse light source for the measurements (diameter of the sampling port, 6 mm), and to gather all the radiation reflected back by the sample, thus approximating the ideal Lambertian reflection condition. The output light is sent through another optical fiber to a laptop-controlled spectrometer (AvaSpec-2048 X 14-USB2) that generates the final pattern in the 248–1050 nm range, at 2.4 nm resolution, through the comparison with the reference reflectance of a factory-calibrated Spectralon^®^ (Labsphere, North Sutton, NH, USA). The preparation of samples for DRS first includes the mixing of the nanoparticle powder (approx. 20 mg) with 480 mg of BaSO_4_, an utterly reflective compound, in order to achieve a very low absorption. The specimen is then introduced into a sample holder, deep enough to approximate the reflectance spectra to that of an infinitely thick sample (R∞). In the measurements of zinc-containing nanoparticles, a total of five 5 s scans were acquired and averaged.

*Vicia faba* seeds from the Botanical Gardens of Rome Tor Vergata were used for plant growth, cytogenetic, and cytotoxicity experiments. Plant growth and germination experiments were conducted as previously described [89]. Briefly, faba bean seeds were sterilized with 5% sodium hypochlorite, washed with sterile water, and soaked for 24 h at 4 °C in the dark before planting in sterile Microbox vessels filled with agarized Murashige and Skoog culture medium (MS, pH 5.8). MS was enriched with 50 and 100 mg kg^−1^ of Zn5@inu, Zn5, Zn5 + inulin, or pure inulin, taking into consideration the inulin coverage of the Zn5@inu sample. For inulin alone, this corresponds to a 0.3 and 0.6% weight for the chosen treatment dose. Pure MS was used for the controls (CNT). A total of 25 Microboxes (each containing five seeds) were cultured at 22 °C for 21 days with a 14 h photoperiod (120 µmol m^−2^ s^−1^). Plants were than uprooted, their roots washed with distilled water, and the total length of the plants determined (millimeter precision ruler). Germination percentages were calculated as the ratio between germinated seeds and the total number of planted seeds multiplied by 100. Plant samples not analyzed immediately were stored at −80° C. For cytogenetic analysis, sterilized seeds were sown in agarized MS. After 4 days, when the major roots were 2 cm long, the root ends were removed by cutting about 5 mm from the distal end to promote secondary root formation. Simultaneously, seed integuments were removed and seedlings were placed in plastic basins with liquid MS. To maintain the cotyledons on the solution’s surface, a plastic grid was used to support the seeds while keeping the roots completely immersed. Seedlings were housed under these conditions in the dark, as specified in the standard protocol for micronuclei analysis, [124] for four days before the nanoparticles were delivered. Each experimental point consisted of 10 germinated seedlings, including negative and positive controls. During treatment, the root systems were immersed in zinc-containing solutions for 24 to 72 h. The genotoxicity of nanomaterials and inulin was tested at a dose comparable to the maximum exposure in in vitro cells (100 mg kg^−1^). Maleic hydrazide (Merck), a highly carcinogenic herbicide, was employed as a positive control (PC) at a concentration of 10^−4^ M in a water solution for 4 h, followed by a 20 h recovery period in MS. The negative control (CNT) consisted of exposing roots to pure MS. Once treatments had been accomplished, secondary roots were chopped and fixed in Carnoy solution (acetic acid:ethanol, 25:75, *v*:*v*) for 30 min before being transferred to a new fixing solution and maintained overnight at 4 °C. After Feulgen staining, root tips were crushed on microscope slides with 45% (*v*:*v*) acetic acid and permanently mounted with Eukitt (ForLab, Stezzano, Italy). MN frequencies were calculated in 25,000 cells at each experimental point (2500 cells per root tip, for a total of 10 treatments). MN frequency analysis was restricted to proliferating cell populations, as determined by the mitotic index of each examined root tip. Cytotoxicity was assessed using trypan blue staining, whereas loss of cell viability was assessed using the Duan et al. [125] methodology with minimal changes. Plant tissues were immersed in a solution of 1% (*w*/*v*) trypan blue in PBS for 45 min in the dark. After three PBS washes, the samples were gently agitated with 100% ethanol to remove any remaining chlorophyll before being rehydrated in a 50% (*v*/*v*) glycerol solution. Images were taken using an Epson Perfection V700 Photo system, and dark patches were quantified against the total surface using Fiji/ImageJ software (Version 2.14, National Institutes of Health, Bethesda, MD, USA); the results were reported as a percentage. Also, the pigment contents in the leaf samples (chlorophyll *a* (Chl *a*), chlorophyll *b* (Chl *b*), and total carotenoid (Car)) were determined by UV–Vis spectrophotometry as described previously on liquid nitrogen powdered leaf samples after extraction with 80% (*v*/*v*) acetone for 24 h in the dark at 4 °C [89]. Absorbances of the centrifuged supernatants were monitored at 470, 645, and 663 nm with a Varian Cary 50 Bio UV–Vis spectrophotometer (Varian, The Netherlands).

The presence of Zn in plant samples was examined by X-ray fluorescence using an energy-dispersive X-ray fluorescence (ED-XRF) spectrometer (SPECTRO XEPOS HE XRF) that was tailored for heavy elements [89]. The equipment ia outfitted with a Pd/Co alloy X-Ray tube. The results were acquired by the “TurboQuant powders and liquid” technique (XRF Analyzer Pro software, version TurboQuant II) using the SPECTRO process calibration model (a combination of the Fundamental Parameter and Extended Compton scattering models, with mass attenuation coefficient calibration). Every experiment was conducted a minimum of three times, and the outcomes are presented as means ± standard error. One-way analysis of variance (ANOVA) was performed on the data, and the post-hoc lowest standard deviation (LSD) test was used to assess the statistical differences (*p* values: * *p* < 0.05; ** *p* < 0.01; *** *p* < 0.001). PAST software, version 4.03, was utilized for this purpose. The Mann–Whitney U non-parametric test was used for the cytogenetic analysis, and values of * *p* < 0.05 were regarded as significant.

### 3.2. Synthetic Procedures

#### 3.2.1. Two-Step Synthesis

The two-step procedure foresees the synthesis of the ZnO NPs by precipitation and ensuing the addition of inulin at 3% weight, according to the following reaction:

$${\mathrm{Zn}({\mathrm{NO}}_{3})}_{2}+2\mathrm{NaOH}\;\overset{T_{P}}{\to }{\mathrm{Zn}(\mathrm{OH})}_{2(s)\;},\;\mathrm{Zn}O_{\left(s\right)}+{2\mathrm{NaNO}}_{3(\mathrm{aq})}\begin{array}{c} \overset{\mathrm{Inulin}}{\to }\\ T_{D} \end{array}\;\mathrm{Zn}{O@\mathrm{inu}}_{\left(s\right)}+H_{2}O+{2\mathrm{NaNO}}_{3(\mathrm{aq})}$$

where T_P_ and T_D_ are the precipitation and digestion temperatures, respectively. In more detail, 0.1 M solutions of ${\mathrm{Zn}({\mathrm{NO}}_{3})}_{2}$ and NaOH are prepared by dissolving suited amounts of reagents in deionized water. A total of 100 mL of NaOH is added dropwise to 100 mL of Zn(NO_3_)_2_ solution previously heated to the selected temperature (see Table 5), kept constant by immersion of the flask in an oil bath. A fine milky precipitate forms immediately and the ensuing suspension is kept under stirring for 24 h to allow digestion of the precipitate. Afterwards, the solution is split into two portions. The first aliquot is centrifuged at 3000 rpm for 10 min and the precipitate is rinsed with deionized water. The procedure is repeated 3 times prior to the final drying at T_D_ temperature that yields a white precipitate. The second aliquot is added with a previously prepared dispersion of inulin in deionized water (3% *w*/*v*) and left under stirring at T_D_ temperature for another 24 h. The resulting suspension is centrifuged at 3000 rpm for 10 min and the precipitate is rinsed with deionized water. This procedure is also repeated 3 times. Eight sets of samples are synthesized by varying the T_P_ and T_D_ temperatures according to the values reported in Table 5. Precipitation and digestion temperatures have been chosen on the basis of our previous studies on the low-temperature synthesis of ZnO nanoparticles in order to assure precipitation of ZnO nanoparticles and the preservation of their properties during the digestion step [24,89].

#### 3.2.2. Gel-like Synthesis

The starting suspension of inulin (concentration 1% *w*/*v*) is prepared by dissolving weighed quantities of inulin into 50 mL of deionized water, through stirring at room temperature for 24 h. After the suspension of the polymer is complete (and forming a sort of gel), solid Zn(NO_3_)_2_·6H_2_O is added to achieve the target concentration of 0.1 M. The solution is stirred for 2 h at room temperature and is then thermalized at 40 °C in an oil bath under vigorous swirling for a further 2 h. Next, 50 mL of NaOH aqueous solution is added at the same concentration (1:1 *w*/*v* ratio) dropwise and the solution is left under stirring overnight at 40 °C. The ZnO@inu nanocomposite is formed during the subsequent centrifugation phase that is performed for 10 min at 3500 rpm and 25 °C. The achieved nanocomposite is washed once with deionized water and dehydrated at 50 °C for 24 h. The sample obtained with this procedure is labelled ZnOInuOP.

## 4. Conclusions

In the present paper, we investigated the preparation procedures of inulin-coated ZnO NPs to tune the synthetic parameters for biostimulation applications. Subsequent characterizations provided information on the chemico-physical properties. The XRD study highlighted that all the samples synthesized and dried at temperatures greater than 40 °C, regardless of the synthetic strategy, contained pure zinc oxide, as previously reported, and that preparation at lower temperatures yielded zinc hydroxide nanoparticles. The infrared spectroscopy investigation confirmed the purity of the samples and reveals that the NPs were successfully coated with inulin, as it is highlighted by the presence of the fructan vibrational fingerprint. However, the binding mechanism was different in one of the cases, i.e., for sample Zn5@inu, prepared at T_P_ = 60, T_D_ = 40 °C, since the IR spectrum indicated the formation of Zn complexes. The SEM microscopy analysis showed that all ZnO-based samples possess nanometric sizes, whereas Zn(OH)_2_ is micrometrical. Also, the one-pot procedure led to the formation of micrometric aggregates of small nanoparticles; therefore, this is less well suited for a homogeneous application in priming protocols. The coating, instead, was most homogeneous and likely did not undergo a denaturation procedure for sample Zn5@inu. The diffuse reflectance study endorsed the role of coating in modulating the material bandgap and shows that the nanocomposites prepared with the two-step procedure had similar values in the 3.25–3.30 eV range. In contrast, the sample prepared with the one-pot reaction pathway showed a remarkably smaller energy difference between the ground and excited states, decreasing to 3.0 eV, likely because of the formation of lower energy defect states within the gap. The observed electronic behavior complied satisfactorily with the structural properties highlighted in the SEM study, which hinted at the presence of quite large micrometric aggregates of small nanometric particles (diameter 30 nm), externally coated by the inulin in the one-pot synthesis sample. Our study indicated that the preparation carried out in two steps at T_P_60, T_D_40 °C yields the most homogeneous and uniformly coated nanoparticles, with electronic properties in line with their uncoated counterparts; hence, they are more suited for biostimulation applications. Proof-of-principle assays employing the latter sample, both coated and uncoated, indicated an effective growth stimulation of *Vicia faba* seedlings upon seed priming, which was more pronounced at doses of 100 mgKg^−1^. The biostimulation worked better when using inulin-coated particles and concomitant Zn accumulation in the leaves was observed. No inherent cyto- or genotoxicty was observed. All in all, the inulin coating played the role of preferential carrier of the ZnO NPs. Given these very encouraging premises, additional studies on other types of coating are in progress, with the aim of further improving the nanoparticle biostimulating effect while retaining the electronic features of the material, as well as its overall formulation chain. Among the many possible future applications, conceivable for our systems, we envisage a prominent role in the manufacturing of a new, low-cost, and marketable class of zinc-nanoparticle-based fertilizers with enhanced features, going from the ready absorption from plant root tissues to the transport through the plant stem up to the leaves. The use of highly bioavailable forms of ZnO like those constituting our nanoparticles, being the oxide compound with the highest metal content among all zinc compounds administered in agriculture, may give a significant boost in the treatment of zinc-deficient soils and plants, and finally increase food and nutrition security.

## Figures and Tables

**Table 1:** IR bands of selected samples and their assignments.

Zn1	Zn1@Inu	Inulin	ZnOInuOP	Zn5	Zn5@Inu	Assignment
401	401		400	400	400	ν _as Zn–O_
482	482					ν _as Zn–O_
507	507					
		592			619	ρ_-CH2_
			626		640	ρ_-CH2_
					665	ρ_-CH2_
			704		696	ρ_-CH2_
716	716					δ_-OH_
			731			
					768	ρ_-CH2_
779	779					
					787	ρ_-CH2_
		827	831		829	2-ketose
851	851					
		866	875		889	Anomeric δ_(C1–H)_, ring vibration _(2-ketofuranose)_
		1016				ν _as C–O–C_
1034	1034		1034		1037	ν _as C–O–C,_ ν _as Zn–O-Zn_
1088	1088				1086	ν _as C–O,_ ν _as Zn–O-Zn_
		1109				
			1134		1132	ν _as C–C_
		1219			1205	β_O-H_
		1269				β_O-H_, δ_C–H_
					1300	β_O-H_
		1330				δ_C–H_, δ_H-C–H_
		1418	1394			δ_C–H_, δ_H-C–H_
		1454			1435	δ_C–H_
			1508		1508	ν _C=O-Zn-O=C as_
			1556		1551	ν _C=O-Zn-O=C as_
		1653	1649		1649	ν _C=O-Zn-O=C as/_δ_H2O_
					1730	ν _C=O as_
			1980			
		2932	2924			ν _C–H_
3210	3210	3304	3365		3370	ν _O–H_ intermolecular H-bonds

**Table 2:** Average size and composition of the samples.

Sample	Average Size (nm)	Zn (at%)	O (at%) *	C (at%)	Si (at%) *
Zn1	1200 ± 50	49.9	50.1		
Zn1@inu	1200 ± 50	45.0	50.7	5.0	
Coating Zn1@inu			50.2	48.8	
Zn2	62 ± 4	48.9	50.7		0.4
Zn2@inu	63 ± 4	48.8	50.1	0.9	
Coating Zn2@inu			50.2	48.8	1.0
Zn3	60 ± 10	49.6	50.4		
Zn3@inu	60 ± 10	49.3	50.2	0.7	
Zn4	52 ± 5	49.8	50.2		
Zn4@inu	54 ± 4	49.0	50.1	0.9	
Zn5	65 ± 4	50.1	49.9		
Zn5@inu	58 ± 1	48.3	50.4	1.3	
ZnOInuOP	30 ± 10	48.7	50.3	1.0	

* Silicon and additional oxygen may be present as contribution from the sample holder of SiO_2_.

**Table 3:** Weight loss differences and degree of coverage of the coated samples. The degree of coverage is expressed as number of polymeric units per particle, converted to number of units per particle in the most right column.

Sample	Weight Loss Difference	Degree of Coverage	Monomers
Zn1@inu	0.15%	53,867	1,616,000
Zn2@inu	0.41%	431	12,917
Zn3@inu	0.42%	381	11,430
Zn4@inu	0.60%	354	10,629
Zn5@inu	0.72%	590	17,700
ZnInuOP	1.67%	192	5770

Monomeric units per particle in the most right column.

**Table 4:** Estimated NP bandgap from Tauc plots of Kubelka–Munk absorption coefficients.

Sample	Estimated Bandgap (eV)
Zn1@inu	2.61
Zn2@inu	3.25
Zn3@inu	3.28
Zn4@inu	3.25
Zn5@inu	3.27
ZnInuOP	3.00

**Table 5:** Summary of the preparation conditions of the samples.

Sample	T_P_ (°C)	T_D_ (°C)
Zn1	30	
Zn1@inu	30	30
Zn2	60	
Zn2@inu	60	60
Zn3	50	
Zn3@inu	50	40
Zn4	40	
Zn4@inu	40	40
Zn5	60	
Zn5@inu	60	40
ZnInuOP	RT	40 *

* The final drying of this sample was carried out at 50 °C.

## Data Availability

The data presented in this study are available on request from the corresponding author.

## References

[1] Zhao L., Zhou X., Kang Z., Peralta-Videa J.R., Zhu Y.G. (2024). Nano-enabled seed treatment: A new and sustainable approach to engineering climate-resilient crops. Sci. Total Environ..

[2] Salam A., Afridi M.S., Javed M.A., Saleem A., Hafeez A., Khan A.R., Zeeshan M., Ali B., Azhar W., Sumaira (2022). Nano-Priming against Abiotic Stress: A Way Forward towards Sustainable Agriculture. Sustainability.

[3] Nile S.H., Thiruvengadam M., Wang Y., Samynathan R., Shariati M.A., Rebezov M., Nile A., Sun M., Venkidasamy B., Xiao J. (2022). Nano-priming as emerging seed priming technology for sustainable agriculture—Recent developments and future perspectives. J. Nanobiotechnol..

[4] Khalaki M.A., Moameri M., Lajayer B.A., Tess Astatkie T. (2021). Influence of nano-priming on seed germination and plant growth of forage and medicinal plants. Plant Growth Regul..

[5] Nawaz M., Sun J., Shabbir S., Khattak W.A., Ren G., Nie X., Bo Y., Javed Q., Du D., Sonne C. (2023). A review of plants strategies to resist biotic and abiotic environmental stressors. Sci. Total Environ..

[6] Limosani F., Bauer E.M., Cecchetti D., Biagioni S., Orlando V., Pizzoferrato R., Prosposito P., Carbone M. (2021). Top-Down N-Doped Carbon Quantum Dots for Multiple Purposes: Heavy Metal Detection and Intracellular Fluorescence. Nanomaterials.

[7] Valentini F., Ciambella E., Boaretto A., Rizzitelli G., Carbone M., Conte V., Cataldo F., Russo V., Casari C.S., Chillura-Martino D.F. (2016). Sensor Properties of Pristine and Functionalized Carbon Nanohorns. Electroanalysis.

[8] Azzouz A., Hejji L., Kumar V., Kim K.H. (2023). Nanomaterials-based aptasensors: An efficient detection tool for heavy-metal and metalloid ions in environmental and biological samples. Environ. Res..

[9] Luo Y., Guo Y. (2023). Nanomaterials for fluorescent detection of vitamin B2: A review. Anal. Biochem..

[10] Anusuyadevi K., Velmathi S. (2023). Design strategies of carbon nanomaterials in fluorescent sensing of biomolecules and metal ions—A review. Results Chem..

[11] Geleta G.S. (2024). Recent advances in electrochemical sensors based on MIPS and nanomaterials for detection of ascorbic dopamineand uric acid—A review. Sens. Bio-Sens. Res..

[12] Gowthaman N.S.K., Amalraj M., Kesavan S., Rajalakshmi K., Shankar S., Sinduja B., Arul P., Karthikeyan R., Loganathan C., Mangala Gowri V. (2023). Zero-, one- and two-dimensional carbon nanomaterials as low-cost catalysts in optical and electrochemical sensing of biomolecules and environmental pollutants. Microchem. J..

[13] Jose J., Prakash P., Jeyaprabha B., Abraham R., Mathew R.M., Zacharia E.S., Thomas V., Thomas J. (2023). Principle, design, strategies, and future perspectives of heavy metal ion detection using carbon nanomaterial-based electrochemical sensors: A review. J. Iran. Chem. Soc..

[14] Kateshiya M.R., Desai M.L., Malek N.I., Kailasa S.K. (2023). Advances in Ultra-small Fluorescence Nanoprobes for Detection of Metal Ions, Drugs, Pesticides and Biomarkers. J. Fluoresc..

[15] Mahajan M.R., Patil P.O. (2022). Design of zero-dimensional graphene quantum dots based nanostructures for the detection of organophosphorus pesticides in food and water: A review. Inorg. Chem. Commun..

[16] Malik S., Singh J., Goyat R., Saharan Y., Chaudhry V., Umar A., Ibrahim A.A., Akbar S., Ameen S., Baskoutas S. (2023). Nanomaterials-based biosensor and their applications: A review. Heliyon.

[17] Wang Z., Yao B., Xiao Y., Tian X., Wang Y. (2023). Fluorescent Quantum Dots and Its Composites for Highly Sensitive Detection of Heavy Metal Ions and Pesticide Residues: A Review. Chemosensors.

[18] Hu X., Li Y., Cao P., Li P., Xing X., Yu Y., Guo R., Yang H. (2023). Recent Advances of Graphene Quantum Dots in Chemiresistive Gas Sensors. Nanomaterials.

[19] Zúñiga K., Rebollar G., Avelar M., Campos-Terán J., Torres E. (2022). Nanomaterial-Based Sensors for the Detection of Glyphosate. Water.

[20] Carbone M., Aneggi E., Figueredo F., Susmel S. (2022). NiO-Nanoflowers Decorating a Plastic Electrode for the Non-Enzymatic Amperometric Detection of H_2_O_2_ in Milk: Old Issue, New Challenge. Food Control.

[21] Gontrani L., Donia D.T., Bauer E.M., Tagliatesta P., Carbone M. (2023). Novel Synthesis of Zinc Oxide Nanoparticles from Type IV Deep Eutectic Solvents. Inorg. Chim. Acta.

[22] Carbone M. (2016). Cu Zn Co Nanosized Mixed Oxides Prepared from Hydroxycarbonate Precursors. J. Alloys Compd..

[23] Carbone M., Piancastelli M.N., Casaletto M.P., Zanoni R., Besnard-Ramage M.J., Comtet G., Dujardin G., Hellner L. (1999). Phenol adsorption on Si(111)7×7 studied by synchrotron radiation photoemission and photodesorption. Surf. Sci..

[24] Donia D.T., Bauer E.M., Missori M., Roselli L., Cecchetti D., Tagliatesta P., Gontrani L., Carbone M. (2021). Room Temperature Syntheses of ZnO and Their Structures. Symmetry.

[25] Aliannezhadi M., Mirsanai S.Z., Jamali M., Shariatmadar Tehrani F. (2024). Optical and structural properties of bare MoO_3_ nanobelt, ZnO nanoflakes, and MoO_3_/ZnO nanocomposites: The effect of hydrothermal reaction times and molar ratios. Opt. Mater..

[26] Bharathi P., Harish S., Shimomura M., Mohan M.K., Archana J., Navaneethan M. (2024). Ultrasensitive and reversible NO_2_ gas sensor based on SnS_2_/TiO_2_ heterostructures for room temperature applications. Chemosphere.

[27] Duan C., Chen G., Wang Z., Li H., Zhang Z., Liu Y., Lu M. (2024). An ultra-sensitive electrochemical sensing platform based on nanoflower-like Au/ZnO array on carbon cloth for the rapid detection of the nitrite residues in food samples. Food Chem..

[28] Li X., Jia F., Luo N., Cai H., Chen J., Ren W., Cheng J., Xu J. (2024). Self-assembly tourmaline@BiFeO_3_ composites with enhanced polarization for dual-selective C_3_H_6_O and H_2_S detection. Sens. Actuators B Chem..

[29] Lv M.S., Li Y.N., Chen G.L., Gao R., Zhang X.F., Deng Z.P., Xu Y.M., Huo L.H., Gao S. (2024). Mesoporous In_2_O_3_/ZnO heterogeneous microtubes replicated from waste willow catkins for high response and rapid detection of NO_2_ gas at low temperature. Sens. Actuators B Chem..

[30] Malik S.B., Mejia-Centeno K.V., Martínez-Alanis P.R., Cabot A., Güell F., Annanouch F.E., Llobet E. (2024). Synergistic effect of CeO_2_ nanoparticles and WO_3_ nanowires in gas sensing applications. Sens. Actuators B Chem..

[31] Murugesan T., Kumar R.R., Ranjan A., Lu M.Y., Lin H.N. (2024). Fabrication of large-area Au nanoparticle decorated ZnO@ZIF-8 core-shell heterostructure nanorods for ppb-level NO_2_ gas sensing at room temperature. Sens. Actuators B Chem..

[32] Pandey G., Bhardwaj M., Kumar S., Lawaniya S.D., Kumar M., Dwivedi P.K., Awasthi K. (2024). Synergistic effects of Pd-Ag decoration on SnO/SnO_2_ nanosheets for enhanced hydrogen sensing. Sens. Actuators B Chem..

[33] Proença M., Rodrigues M.S., Moura C., Machado A.V., Borges J., Vaz F. (2024). Nanoplasmonic Au:CuO thin films functionalized with APTES to enhance the sensitivity of gas sensors. Sens. Actuators B Chem..

[34] Renganathan B., Gopakumar C.K., Priya A.K., Rao S.K., Sastikumar D., Silambarasan M., Kannapiran N. (2024). Optimizing Gas Sensing Performance of CuO Nanoparticles via Sol-Gel Synthesis Approach for Efficient Detection of Ammonia Gas. Mater. Res. Bull..

[35] Carbone M., Missori M., Micheli L., Tagliatesta P., Bauer E.M. (2020). NiO Pseudocapacitance and Optical Properties: Does the Shape Win?. Materials.

[36] Carbone M. (2018). Zn Defective ZnCo_2_O_4_ Nanorods as High Capacity Anode for Lithium Ion Batteries. J. Electroanal. Chem..

[37] Gontrani L., Bauer E.M., Talone A., Missori M., Imperatori P., Tagliatesta P., Carbone M. (2023). CuO Nanoparticles and Microaggregates: An Experimental and Computational Study of Structure and Electronic Properties. Materials.

[38] BhaskaraRao B.V., Pabba D.P., Aepuru R., Akbari-Fakhrabadi A., Lokhande P., Udayabhaskar R., Rosales-Vera M., Espinoza-González R. (2023). Fe_3_O_4_ nanoparticles intercalated reduced graphene oxide nanosheets for supercapacitor and lithium-ion battery anode performance. J. Mater. Sci. Mater. Electron..

[39] Bhaviripudi V.R., Dwivedi P.K., Pabba D.P., Aepuru R., Nakate U.T., Espinoza-González R., Shelke M.V. (2023). Evaluation of Fe_3_O_4_ incorporated functionalized carbon nanotube self-standing buckypaper as electrodes for solid-state symmetric supercapacitor. J. Energy Storage.

[40] Jalilzadeh H., Outokesh M., Shafiekhani A., Hosseinpour M., Tayyebi A. (2023). Magnetite nanoparticles embedded on reduced graphene oxide as an anode material for high capacity and long cycle-life Li-ion battery. J. Energy Storage.

[41] Lv H., Xiao Z., Zhai S., Wang X., Hao J., An Q. (2023). Designed formation of C/Fe_3_O_4_@Ni(OH)_2_ cathode with enhanced pseudocapacitance for asymmetric supercapacitors. J. Colloid Interface Sci..

[42] Park S., Ji S., Kim S.K., Yoon Y., Yim S., Song W., Myung S., Lee S.S., An K.S. (2023). Architectural engineering of vertically expanded graphene-CoMn_2_O_4_ compounds based interdigital electrode for in-plane micro-supercapacitor. J. Alloys Compd..

[43] Patil S.L., Pawar O.Y., Patil H.S., Sutar S.S., Kamble G.U., Kim D.K., Kim J.H., Kim T.G., Kamat R.K., Dongale T.D. (2023). The g-C_3_N_4_-TiO_2_ nanocomposite for non-volatile memory and artificial synaptic device applications. J. Alloys Compd..

[44] Paunkumar P., Bhuvaneswari C., Priya R.L., Hariprasad B.S., Dhayanithi C.A., Babu S.G. (2024). Functionalized Nanomaterials as Supercapacitor Devices: Current Trends and Beyond. Materials Horizons: From Nature to Nanomaterials.

[45] Wang K., Cao J., Gao J., Zhao J., Jiang W., Ahmad W., Jiang J., Ling M., Liang C., Chen J. (2023). Unveiling the structure-activity relationship of hollow spindle-like α-Fe_2_O_3_ nanoparticles via phosphorus doping engineering for enhanced lithium storage. SM&Ts.

[46] Wang Q., Chen Z., Bai S., Wang X., Zhang Y. (2023). Innovative BVO@CuO design: A high-performance vanadium-based anode material for Li-ion batteries. J. Alloys Compd..

[47] Wei J., Hu F., Shen X., Chen B., Chen L., Wang Z., Lv C., Ouyang Q. (2023). Defective core–shell NiCo_2_S_4_/MnO_2_ nanocomposites for high performance solid-state hybrid supercapacitors. J. Colloid Interface Sci..

[48] Kim J.W., Carbone M., Tallarida M., Dil J.H., Horn K., Casaletto M.P., Flammini R., Piancastelli M.N. (2004). Adsorption of 2,3-butanediol on Si(100). Surf. Sci..

[49] Carbone M., Zanoni R., Piancastelli M.N., Comtet G., Dujardin G., Hellner L. (1996). Synchrotron radiation photoemission and photostimulated desorption of deuterated methanol on Si(111)7 × 7 and Si(100)2 × 1. Surf. Sci..

[50] Bauer E.M., Talone A., Imperatori P., Briancesco R., Bonadonna L., Carbone M. (2023). The Addition of Co into CuO–ZnO Oxides Triggers High Antibacterial Activity and Low Cytotoxicity. Nanomaterials.

[51] Chioibas R., Borcan F., Mederle O., Stoian D., Soica C.M. (2019). Comparative characterization of different samples containing nano-ZnO particles with applicability in topical therapies. Mater. Plast..

[52] Carbone M., Sabbatella G., Antonaroli S., Remita H., Orlando V., Biagioni S., Nucara A. (2015). Exogenous Control over Intracellular Acidification: Enhancement via Proton Caged Compounds Coupled to Gold Nanoparticles. Biochim. Biophys. Acta Gen. Subj..

[53] Hernandez-Delgadillo R., Velasco-Arias D., Martinez-Sanmiguel J.J., Diaz D., Zumeta-Dube I., Arevalo-Niño K., Cabral-Romero C. (2013). Bismuth oxide aqueous colloidal nanoparticles inhibit Candida albicans growth and biofilm formation. Int. J. Nanomed..

[54] Khan M.F., Hameedullah M., Ansari A.H., Ahmad E., Lohani M.B., Khan R.H., Alam M.M., Khan W., Husain F.M., Ahmad I. (2014). Flower-shaped ZnO nanoparticles synthesized by a novel approach at near-room temperatures with antibacterial and antifungal properties. Int. J. Nanomed..

[55] Król A., Pomastowski P., Rafińska K., Railean-Plugaru V., Buszewski B. (2017). Zinc oxide nanoparticles: Synthesis, antiseptic activity and toxicity mechanism. Adv. Colloid Interface Sci..

[56] Kyomuhimbo H.D., Michira I.N., Mwaura F.B., Derese S., Feleni U., Iwuoha E.I. (2019). Silver–zinc oxide nanocomposite antiseptic from the extract of Bidens pilosa. SN Appl. Sci..

[57] Liang H., Wang H., Sun X., Xu W., Meng N., Zhou N. (2023). Development of ZnO/Ag nanoparticles supported polydopamine-modified montmorillonite nanocomposites with synergistic antibacterial performance. Appl. Clay Sci..

[58] Lin W., Fan S., Liao K., Huang Y., Cong Y., Zhang J., Jin H., Zhao Y., Ruan Y., Lu H. (2023). Engineering zinc oxide hybrid selenium nanoparticles for synergetic anti-tuberculosis treatment by combining Mycobacterium tuberculosis killings and host cell immunological inhibition. Front. Cell. Infect. Microbiol..

[59] Obeizi Z., Benbouzid H., Bouarroudj T., Bououdina M. (2021). Excellent antimicrobial and anti-biofilm activities of Fe-SnO_2_ nanoparticles as promising antiseptics and disinfectants. Adv. Nat. Sci. Nanosci. Nanotechnol..

[60] Pomastowski P., Król-Górniak A., Railean-Plugaru V., Buszewski B. (2020). Zinc oxide nanocomposites—Extracellular synthesis, physicochemical characterization and antibacterial potential. Materials.

[61] Sasani M., Fataei E., Safari R., Nasehi F., Mosayebi M. (2021). Antibacterial effects of iron oxide and silver nanoparticles synthesized bbacillus subtilis: A comparative study. Desalination Water Treat..

[62] Balu S.K., Andra S., Jeevanandam J., Kulabhusan P.K., Khamari A., Vedarathinam V., Hamimed S., Chan Y.S., Danquah M.K. (2023). Exploring the potential of metal oxide nanoparticles as fungicides and plant nutrient boosters. Crop Prot..

[63] Deng C., Protter C.R., Wang Y., Borgatta J., Zhou J., Wang P., Goyal V., Brown H.J., Rodriguez-Otero K., Dimkpa C.O. (2023). Nanoscale CuO charge and morphology control Fusarium suppression and nutrient biofortification in field-grown tomato and watermelon. Sci. Total Environ..

[64] Hassan M.U., Kareem H.A., Hussain S., Guo Z., Niu J., Roy M., Saleem S., Wang Q. (2023). Enhanced salinity tolerance in Alfalfa through foliar nano-zinc oxide application: Mechanistic insights and potential agricultural applications. Rhizosphere.

[65] Mandal T.K. (2024). ZnO Nanoparticles: Sustainable Plant Production. Nanotechnology in the Life Sciences.

[66] Mazhar M.W., Ishtiaq M., Maqbool M., Ullah F., Sayed S.R.M., Mahmoud E.A. (2023). Seed priming with iron oxide nanoparticles improves yield and antioxidant status of garden pea (*Pisum sativum* L.) grown under drought stress. S. Afr. J. Bot..

[67] Rabea A., Naeem E., Balabel N.M., Daigham G.E. (2023). Management of potato brown rot disease using chemically synthesized CuO-NPs and MgO-NPs. Bot. Stud..

[68] Filippou P., Tanou G., Molassiotis A., Fotopoulos V. (2012). Plant Acclimation to Environmental Stress Using Priming Agents.

[69] Méndez A.A., Pena L.B., Benavides M.P., Gallego S.M. (2016). Priming with NO controls redox state and prevents cadmium-induced general up-regulation of methionine sulfoxide reductase gene family in Arabidopsis. Biochimie.

[70] Acharya P., Jayaprakasha G.K., Crosby K.M., Jifon J.L., Patil B.S. (2020). Nanoparticle-Mediated Seed Priming Improves Germination, Growth, Yield, and Quality of Watermelons (*Citrullus lanatus*) at multi-locations in Texas. Sci. Rep..

[71] Timmusk S., Seisenbaeva G., Behers L. (2018). Titania (TiO_2_) nanoparticles enhance the performance of growth-promoting rhizobacteria. Sci. Rep..

[72] Mazhar M.W., Ishtiaq M., Maqbool M., Atiq Hussain S., Casini R., Abd-ElGawad A.M., Elansary H.O. (2023). Seed Nano-Priming with Calcium Oxide Maintains the Redox State by Boosting the Antioxidant Defense System in Water-Stressed Carom (*Trachyspermum ammi* L.) Plants to Confer Drought Tolerance. Nanomaterials.

[73] Setty J., Samant S.B., Yadav M.K., Manjubala M., Pandurangam V. (2023). Benefcial efects of bio-fabricated selenium nanoparticles as seed nanopriming agent on seed germination in rice (*Oryza sativa* L.). Sci. Rep..

[74] Yousaf N., Ishfaq M., Qureshi H.A., Saleem A., Yang H., Sardar M.F., Zou C. (2023). Characterization of Root and Foliar-Applied Iron Oxide Nanoparticles (α-Fe_2_O_3_, γ-Fe_2_O_3_, Fe_3_O_4_, and Bulk Fe_3_O_4_) in Improving Maize (*Zea mays* L.) Performance. Nanomaterials.

[75] Rai-Kalal P., Jajoo A. (2021). Priming with zinc oxide nanoparticles improve germination and photosynthetic performance in wheat. Plant Physiol. Biochem..

[76] Donia D.T., Carbone M. (2023). Seed Priming with Zinc Oxide Nanoparticles to Enhance Crop Tolerance to Environmental Stresses. Int. J. Mol. Sci..

[77] Zafar S., Perveen S., Khan M.K., Shaheen M.R., Hussain R., Sarwar N., Rashid S., Nafees M., Farid G., Alamri S. (2022). Effect of zinc nanoparticles seed priming and foliar application on the growth and physiobiochemical indices of spinach (*Spinacia oleracea* L.) under salt stress. PLoS ONE.

[78] Rizwan M., Ali S., Ali B., Adrees M., Arshad M., Hussain A., Zia ur Rehman M., Waris A.A. (2019). Zinc and iron oxide nanoparticles improved the plant growth and reduced the oxidative stress and cadmium concentration in wheat. Chemosphere.

[79] Du W., Yang J., Peng Q., Liang X., Mao H. (2019). Comparison study of zinc nanoparticles and zinc sulphate on wheat growth: From toxicity and zinc biofortification. Chemosphere.

[80] Durgude S.A., Ram S., Kumar R., Singh S.V., Singh V., Durgude A.G., Pramanick B., Maitra S., Gaber A., Hossain A. (2022). Synthesis of Mesoporous Silica and Graphene-Based FeO and ZnO Nanocomposites for Nutritional Biofortification and Sustained the Productivity of Rice (*Oryza sativa* L.). J. Nanomater..

[81] Ghani M.I., Saleem S., Rather S.A., Rehmani M.S., Alamri S., Rajput V.D., Kalaji H.M., Saleem N., Sial T.A., Liu M. (2022). Foliar application of zinc oxide nanoparticles: An effective strategy to mitigate drought stress in cucumber seedling by modulating antioxidant defense system and osmolytes accumulation. Chemosphere.

[82] Rajput V.D., Minkina T.M., Behal A., Sushkova S.N., Mandzhieva S., Singh R., Gorovtsov A., Tsitsuashvili V.S., Purvis W.O., Ghazaryan K.A. (2018). Effects of zinc-oxide nanoparticles on soil, plants, animals and soil organisms: A review. Environ. Nanotechnol. Monit. Manag..

[83] Afsal S., Sharma D., Singh N.K. (2021). Eco-friendly synthesis of phytochemical-capped iron oxide nanoparticles as nano-priming agent for boosting seed germination in rice (*Oryza sativa* L.). Environ. Sci. Pollut. Res..

[84] Mahakham W., Theerakulpisut P., Maensiri S., Phumying S., Sarmah A.K. (2016). Environmentally benign synthesis of phytochemicals-capped gold nanoparticles as nanopriming agent for promoting maize seed germination. Sci. Total Environ..

[85] Song K., Zhao D., Sun H., Gao J., Li S., Hu T., He X. (2022). Green nanopriming: Responses of alfalfa (*Medicago sativa* L.) seedlings to alfalfa extract capped and light-induced silver nanoparticles. BMC Plant Biol..

[86] Kalimuthu R., Sella K.M., Antony D., Rajaprakasam S., Chokkalingam V., Chidambaram P., Kanagarajam S. (2023). Nanopriming Action of Microwave-Assisted Biofunctionalized ZnO Nanoparticles to Enhance the Growth under Moisture Stress in *Vigna radiata*. ACS Omega.

[87] Zungu B., Paumo H.K., Gaorongwe J.L., Tsuene G.N., Ruzvidzo O., Katata-Seru L. (2023). Zn nutrients-loaded chitosan nanocomposites and their efficacy as nanopriming agents for maize (*Zea mays*) seeds. Front. Chem..

[88] Del Buono D., Luzi F., Tolisano C., Puglia D., Di Michele A. (2022). Synthesis of a Lignin/Zinc Oxide Hybrid Nanoparticles System and Its Application by Nano-Priming in Maize. Nanomaterials.

[89] Carbone M., De Rossi S., Donia D.T., Di Marco G., Gustavino B., Roselli L., Tagliatesta P., Canini A., Gismondi A. (2023). Biostimulants promoting growth of *Vicia faba* L. seedlings: Inulin coated ZnO nanoparticles. Chem. Biol. Technol. Agric..

[90] Redondo-Cuenca A., Herrera-Vàzquez S.E., Condezo-Hoyos L., Gómez-Ordóñez E., Rupérez P. (2021). Inulin extraction from common inulin-containing plant sources. Ind. Crops Prod..

[91] Apolinário A.C., de Carvalho E.M., de Lima Damasceno B.P.G., da Silva P.C.D., Converti A., Pessoa A., da Silva J.A. (2017). Extraction, isolation and characterization of inulin from Agave sisalana boles. Ind. Crops Prod..

[92] Waqas Mazhar M., Ishtiaq M., Hussain I., Parveen A., Hayat Bhatti K., Azeem M., Thind S., Ajaib M., Maqbool M., Sardar T. (2022). Seed nano-priming with Zinc Oxide nanoparticles in rice mitigates drought and enhances agronomic profile. PLoS ONE.

[93] Abbas S.F., Bukhari M.A., Raza M.A.S., Abbasi G.H., Ahmad Z., Alqahtani M.D., Almutairi K.F., Abd Allah E.F., Iqbal M.A. (2023). Enhancing Drought Tolerance in Wheat Cultivars through Nano-ZnO Priming by Improving Leaf Pigments and Antioxidant Activity. Sustainability.

[94] El-Bassiouny H.M.S., Mahfouze H.A., Abdallah M.M.S., Bakry B.A., El-Enany M.A.M. (2022). Physiological and Molecular Response of Wheat Cultivars to Titanium Dioxide or Zinc Oxide Nanoparticles under Water Stress Conditions. Int. J. Agron..

[95] Al-Salama Y. (2022). Effect of Seed Priming with ZnO Nanoparticles and Saline Irrigation Water in Yield and Nutrients Uptake by Wheat. Plants Environ. Sci. Proc..

[96] Salam A., Khan A.R., Liu L., Yang S., Azhar W., Ulhassan Z., Zeeshan M., Wu J., Fan X., Gan Y. (2022). Seed priming with zinc oxide nanoparticles downplayed ultrastructural damage and improved photosynthetic apparatus in maize under cobalt stress. J. Hazard. Mater..

[97] Wang W., Yamaji N., Ma J.F. (2019). Molecular Mechanism of Cadmium Accumulation in Rice. Cadmium Toxicity. Current Topics in Environmental Health and Preventive Medicine.

[98] Kashyap D., Siddiqui Z.A. (2020). Effect of different inocula of *Meloidogyne incognita* and *Pseudomonas syringae* pv. *pisi* with and without *Rhizobium leguminosarum* on growth, chlorophyll, carotenoid and proline contents of pea. Indian Phytopathol..

[99] Li M., Ahammed G.J., Li C., Bao X., Yu J., Huang C., Yin H., Zhou J. (2016). Brassinosteroid Ameliorates Zinc Oxide Nanoparticles-Induced Oxidative Stress by Improving Antioxidant Potential and Redox Homeostasis in Tomato Seedling. Front. Plant Sci..

[100] Bauer E.M., Bogliardi G., Ricci C., Cecchetti D., De Caro T., Sennato S., Nucara A., Carbone M. (2022). Syntheses of APTMS-Coated ZnO: An Investigation towards Penconazole Detection. Materials.

[101] Aminoff G. (1921). XXIV. Über Lauephotogramme und Struktur von Zinkit. Z. Krist.—Cryst. Mater..

[102] Stahl R., Jung C., Lutz H.D., Kockelmann W., Jacobs H. (1998). Kristallstrukturen und Wasserstoffbrückenbindungen bei β-Be(OH)2 und ϵ-Zn(OH)2. Z. Anorg. Allg. Chem..

[103] Ghotbi M.Y. (2010). Synthesis and characterization of nano-sized ɛ-Zn(OH)_2_ and its decomposed product, nano-zinc oxide. J. Alloys Compd..

[104] Top A., Çetinkaya H. (2015). Zinc oxide and zinc hydroxide formation via acquous precipitation: Effect of the preparation route and lysozyme addition. Mater. Chem. Phys..

[105] Akram W., Garud N. (2020). Optimization of inulin production process parameters using response surface methodology. Future J. Pharm. Sci..

[106] Salavati-Niasari M., Gholami-Daghian M., Esmaeili-Zare M., Sangsefidi F.S. (2013). Solid State Synthesis and Characterization of Zinc Oxide (ZnO) Microflakes by [Bis(acetylacetonato)zinc(II)] and Sodium Hydroxide at Room Temperature. J. Clust. Sci..

[107] Rauf N., Ilyas S., Heryanto H., Rahmat R., Fahri A.N., Rahmi M.H., Tahir D. (2021). The Correlation between Structural and Optical Properties of Zinc Hydroxide Nanoparticle in Supports Photocatalytic Performance. Opt. Mater..

[108] Tian H., Ghorbanpour M., Kariman K. (2018). Manganese oxide nanoparticle-induced changes in growth, redox reactions and elicitation of antioxidant metabolites in deadly nightshade (*Atropa belladonna* L.). Ind. Crops Prod..

[109] Huang D., Dang F., Huang Y., Chen N., Zhou D. (2022). Uptake, translocation, and transformation of silver nanoparticles in plants. Environ. Sci. Nano.

[110] Kubelka P., Munk F. (1931). Ein Beitrag Zur Optik Der Farbanstriche. Z. Tech. Phys..

[111] Missori M., Pulci O., Teodonio L., Violante C., Kupchak I., Bagniuk J., Łojewska J., Mosca Conte A. (2014). Optical response of strongly absorbing inhomogeneous materials: Application to paper degradation. Phys. Rev. B.

[112] Missori M. (2016). Optical spectroscopy of ancient paper and textiles. Riv. Nuovo Cimento.

[113] Smith R.A. (1978). Semiconductors.

[114] Özgür Ü., Alivov Y.I., Liu C., Teke A., Reshchikov M.A., Doğan S., Avrutin V., Cho S.-J., Morkoç H. (2005). A comprehensive review of ZnO materials and devices. J. Appl. Phys..

[115] Islam S.M., Gayen T., Moussawi A., Shi L., Seredych M., Bandosz T.J., Alfano R. (2013). Structural and optical characterization of Zn(OH)_2_ and its composites with graphite oxides. Opt. Lett..

[116] Brus L.E. (1984). Electron–electron and electron-hole interactions in small semiconductor crystallites: The size dependence of the lowest excited electronic state. J. Chem. Phys..

[117] Repp S., Erdem E. (2016). Controlling the exciton energy of zinc oxide (ZnO) quantum dots by changing the confinement conditions. Spectrochim. Acta A.

[118] Xie X., Mao C., Liu X., Tan L., Cui Z., Yang X., Zhu S., Li Z., Yuan X., Zheng Y. (2018). Tuning the Bandgap of Photo-Sensitive Polydopamine/Ag_3_PO_4_/Graphene Oxide Coating for Rapid, Noninvasive Disinfection of Implants. ACS Cent. Sci..

[119] Guan S., Cheng Y., Hao L., Yoshida H., Chiaki T., Tianzhuo Z., Takaomi I., Tangbin Q., Lu Y. (2023). Oxygen vacancies induced band gap narrowing for efficient visible-light response in carbon-doped TiO_2_. Sci. Rep..

[120] Ayoub I., Kumar V., Abolhassani R., Sehgal R., Sharma V., Sehgal R., Swart H.C., Mishra Y.K. (2022). Advances in ZnO: Manipulation of defects for enhancing their technological potentials. Nanotechnol. Rev..

[121] Cai L., Song A.Y., Li W., Hsu P.C., Lin D., Catrysse P.B., Liu Y., Peng Y., Chen J., Wang H. (2018). Spectrally Selective Nanocomposite Textile for Outdoor Personal Cooling. Adv. Mater..

[122] van der Leij M. (1978). The possibility of black zinc oxide as spectrally selective coating for lower temperature solar collectors. J. Electrochem. Soc..

[123] Hu P., An J., Faulkner M.M., Wu H., Li Z., Tian X., Giraldo J.P. (2020). Nanoparticle Charge and Size Control Foliar Delivery Efficiency to Plant Cells and Organelles. ACS Nano.

[124] Gustavino B., Carboni G., Petrillo R., Paoluzzi G., Santovetti E., Rizzoni M. (2016). Exposure to 915 MHz radiation induces micronuclei in *Vicia faba* root tips. Mutagenesis.

[125] Duan Y., Zhang W., Li B., Wang Y., Li K., Sodmergen, Han C., Zhang Y., Li X. (2010). An endoplasmic reticulum response pathway mediates programmed cell death of root tip induced by water stress in Arabidopsis. New Phytol..

